# Association Between Social Media Use and Self-reported Symptoms of Depression in US Adults

**DOI:** 10.1001/jamanetworkopen.2021.36113

**Published:** 2021-11-23

**Authors:** Roy H. Perlis, Jon Green, Matthew Simonson, Katherine Ognyanova, Mauricio Santillana, Jennifer Lin, Alexi Quintana, Hanyu Chwe, James Druckman, David Lazer, Matthew A. Baum, John Della Volpe

**Affiliations:** 1 Massachusetts General Hospital, Boston; 2 Harvard Medical School, Boston, Massachusetts; 3 Northeastern University, Boston, Massachusetts; 4 University of Pennsylvania, Philadelphia; 5 Rutgers University, New Brunswick, New Jersey; 6 Boston Children’s Hospital, Boston, Massachusetts; 7 Northwestern University, Evanston, Illinois; 8 Harvard University, Cambridge, Massachusetts

## Abstract

**Importance:**

Some studies suggest that social media use is associated with risk for depression, particularly among children and young adults.

**Objective:**

To characterize the association between self-reported use of individual social media platforms and worsening of depressive symptoms among adults.

**Design, Setting, and Participants:**

This survey study included data from 13 waves of a nonprobability internet survey conducted approximately monthly between May 2020 and May 2021 among individuals aged 18 years and older in the US. Data were analyzed in July and August 2021.

**Main Outcomes and Measures:**

Logistic regression was applied without reweighting, with a 5 point or greater increase in 9-item Patient Health Questionnaire (PHQ-9) score as outcome and participant sociodemographic features, baseline PHQ-9, and use of each social media platform as independent variables.

**Results:**

In total, 5395 of 8045 individuals (67.1%) with a PHQ-9 score below 5 on initial survey completed a second PHQ-9. These respondents had a mean (SD) age of 55.8 (15.2) years; 3546 respondents (65.7%) identified as female; 329 respondents (6.1%) were Asian, 570 (10.6%) Black, 256 (4.7%) Hispanic, 4118 (76.3%) White, and 122 (2.3%) American Indian or Alaska Native, Pacific Islander or Native Hawaiian, or other. Among eligible respondents, 482 (8.9%) reported 5 points or greater worsening of PHQ-9 score at second survey. In fully adjusted models for increase in symptoms, the largest adjusted odds ratio (aOR) associated with social media use was observed for Snapchat (aOR, 1.53; 95% CI, 1.19-1.96), Facebook (aOR, 1.42; 95% CI, 1.10-1.81), and TikTok (aOR, 1.39; 95% CI, 1.03-1.87).

**Conclusions and Relevance:**

Among survey respondents who did not report depressive symptoms initially, social media use was associated with greater likelihood of subsequent increase in depressive symptoms after adjustment for sociodemographic features and news sources. These data cannot elucidate the nature of this association, but suggest the need for further study to understand how social media use may factor into depression among adults.

## Key Points

**Question:**

Is social media use by adults associated with subsequent increases in depressive symptoms?

**Findings:**

In this survey study, 5395 individuals with minimal depressive symptoms on initial survey who reported use of Snapchat, Facebook, or TikTok were more likely to report increased levels of depressive symptoms on a later survey.

**Meaning:**

These results suggest that certain social media use preceded worsening of depressive symptoms.

## Teaser

This survey study of US adults examines increases in depressive symptoms following use of social media apps and websites during the COVID-19 pandemic.

## Introduction

Social media use has been associated with diminished well-being and greater levels of anxiety and depression, predominantly in cross-sectional studies among adolescents^1^ or young adults,^2^ although concern has been raised that reporting bias may result from individuals with greater depressive symptoms overreporting social media use.^3^ A small number of short-term longitudinal studies provide further support for this association—for example, among 82 young adults sampled 2 weeks apart.^4,5^

These results have 2 notable gaps that raise questions. First, is the observed cross-sectional relationship also apparent in longitudinal studies, as suggested by a randomized trial of Facebook discontinuation?^6^ Second, does this risk apply to older consumers of social media? To investigate these questions, we used data from multiple waves of an ongoing 50-state US survey.

## Methods

Data were drawn from 13 waves of a nonprobability internet survey deployed via a multipanel commercial vendor approximately every month between May 2020 and May 2021 in individuals aged 18 years and older. The study was approved by the institutional review board of Harvard University, and participants provided written informed consent. Reporting followed the American Association for Public Opinion Research (AAPOR) reporting guideline.

The survey sampling strategy incorporated quotas for sex, age, and race and ethnicity within each state; attention checks and open-ended answers were used to filter out unreliable respondents. Race and ethnicity was self-reported from 5 census categories to confirm representativeness of the US population. Participants also completed the 9-item Patient Health Questionnaire (PHQ-9).^7^ They were asked, “Do you ever use any of the following social media sites or apps?”; we focused a priori on Facebook, Instagram, LinkedIn, Pinterest, TikTok, Twitter, Snapchat, and YouTube. They were further asked to identify any sources of COVID-19 related news they consumed in the past 24 hours (ie, cable television, network television, or news website), which we used as a proxy for news sources more generally, while examining web-based vs television-based news separately; number of social supports available “to talk to if you had a problem, felt sad, or depressed”; and face-to-face meetings with nonhousehold members in the prior 24 hours. As some survey modules were randomly assigned to a subset of participants in each wave to diminish overall survey length, 1646 returning participants completed news source questions, and 4808 completed social supports questions (Table 1).

**Table 1.** Comparison of Survey Respondents Based on Completion of Both PHQ-9 Surveys

Characteristic	Participants, No. (%)			*P* value
	No follow-up (n = 2650)	Follow-up survey (n = 5395)	Total (N = 8045)	
Age, y				
Mean (SD), y	52.6 (16.2)	55.8 (15.2)	54.8 (15.6)	<.001
≥35	2195 (82.8)	4766 (88.3)	6961 (86.5)	<.001
Sex				
Women	1766 (66.6)	3546 (65.7)	5312 (66.0)	.42
Men	884 (33.4)	1849 (34.3)	2733 (34.0)	
Race/ethnicity				
Asian American	175 (6.6)	329 (6.1)	504 (6.3)	.13
Black	281 (10.6)	570 (10.6)	851 (10.6)	
Hispanic	118 (4.5)	256 (4.7)	374 (4.6)	
White	1992 (75.2)	4118 (76.3)	6110 (75.9)	
Other^a^	84 (3.2)	122 (2.3)	206 (2.6)	
Household income, mean (SD), $	70 900 (66 700)	68 300 (63 900)	69 200 (64 800)	.10
Some college education	1245 (47.0)	2516 (46.6)	3761 (46.7)	.77
Region				
Rural	390 (14.7)	894 (16.6)	1284 (16.0)	.10
Suburban	1637 (61.8)	3258 (60.4)	4895 (60.8)	
Urban	623 (23.5)	1243 (23.0)	1866 (23.2)	
No. of social supports, mean (SD)^b^	3.7 (3.03)	3.7 (3.0)	3.7 (3.0)	.29
No. of face-to-face interactions, mean (SD)^c^	4.8 (11.3)	4.2 (10.3)	4.4 (10.7)	.01
News source^d^				
Internet	194 (31.0)	473 (28.7)	667 (29.3)	.28
Television	460 (73.5)	1204 (73.0)	1664 (73.1)	.81
Social media platform use				
Facebook	2028 (76.5)	4050 (75.1)	6078 (75.6)	.15
Instagram	949 (35.8)	1682 (31.2)	2631 (32.7)	<.001
LinkedIn	576 (21.7)	1128 (20.9)	1704 (21.2)	.39
Pinterest	882 (33.3)	1716 (31.8)	2598 (32.3)	.18
TikTok	266 (10.0)	387 (7.2)	653 (8.1)	<.001
Twitter	663 (25.0)	1267 (23.5)	1930 (24.0)	.13
Snapchat	458 (17.3)	698 (12.9)	1156 (14.4)	<.001
YouTube	1642 (62.0)	3135 (58.1)	4777 (59.4)	<.001
Baseline PHQ-9 total, mean (SD)	1.36 (1.44)	1.29 (1.43)	1.31 (1.43)	.04
Clinically significant increase (PHQ-9)	NA	482 (8.9)	482 (8.9)	NA

Abbreviation: PHQ-9, 9-item Patient Health Questionnaire.

^a^ Other refers to self-report of American Indian or Alaska Native, Pacific Islander or Native Hawaiian, or other, based upon US Census categories.

^b^ Social support missing data in 245 nonreturning participants, 587 returning.

^c^ Missing data in 10 nonreturning participants, 25 returning.

^d^ News source data missing in 2024 nonreturning participants, 3745 returning.

### Statistical Analysis

The study cohort included participants who completed the PHQ-9 in at least 2 survey waves with a PHQ-9 total score less than 5 (ie, less than mild depression) at the index survey. We applied logistic regression for participants with a 5 or more point increase in PHQ-9 score (ie, the threshold for clinical significance^7^) as outcome, and for sociodemographic features, baseline PHQ-9, and use of each social media platform as independent variables using glm in R version 3.6 (R Project for Statistical Computing) without reweighting. The threshold for statistical significance was set at 2-sided *P* < .05.

## Results

Overall, 5395 of 8045 individuals (67.1%) with a PHQ-9 score below 5 on initial survey completed a subsequent PHQ-9 (Table 1). These respondents had mean (SD) age of 55.8 (15.2) years; 3546 respondents (65.7%) identified as female; 329 respondents (6.1%) were Asian, 570 (10.6%) Black, 256 (4.7%) Hispanic, 4118 (76.3%) White, and 122 (2.3%) American Indian or Alaska Native, Pacific Islander or Native Hawaiian, or other. Mean (SD) PHQ-9 score at initial survey for this group of subsequent responders was 1.29 (1.43). At first follow-up survey, 482 respondents (8.9%) experienced an increase in PHQ-9 score of 5 points or greater.

In adjusted regression models, Snapchat, Facebook, and TikTok use at first survey were significantly associated with greater risk of increase in self-reported depressive symptoms, with adjusted odds ratios (aOR) of 1.53 (95% CI, 1.19-1.96), 1.42 (95% CI, 1.10-1.81), and 1.39 (95% CI, 1.03-1.87), respectively (Table 2). Incorporating terms for television or internet news consumed in the past 24 hours, number of social supports, and number of daily face-to-face interactions on the initial survey did not meaningfully change these associations with the notable exception of Snapchat, where aORs were diminished from 1.53 (95% CI, 1.19-1.96) to 1.12 (95% CI, 0.70-1.81) with the inclusion of terms for news source (Table 2).

**Table 2.** Effect Sizes for Social Media Platform With or Without Adjustment for Social Support, Social Interactions, and News Source

Platform	Full cohort, unadjusted OR (95% CI)^a^	*P* value	aOR (95% CI)		
			With news sources^b^	With social support	With social interactions
Facebook	1.42 (1.10-1.81)	.007	1.38 (0.88-2.19)	1.51 (1.16-1.97)	1.40 (1.09-1.79)
Instagram	1.18 (0.95-1.46)	.13	NA	NA	NA
LinkedIn	1.03 (0.80-1.33)	.24	NA	NA	NA
Pinterest	0.94 (0.76-1.16)	.57	NA	NA	NA
TikTok	1.39 (1.03-1.87)	.03	1.40 (0.78-2.52)	1.41 (1.03-1.94)	1.38 (1.02-1.86)
Twitter	1.05 (0.84-1.31)	.41	NA	NA	NA
Snapchat	1.53 (1.19-1.96)	<.001	1.12 (0.70-1.81)	1.61 (1.23-2.10)	1.52 (1.19-1.96)
YouTube	1.16 (0.93-1.44)	.18	NA	NA	NA

Abbreviations: NA, not applicable; OR, odds ratio.

^a^ Unadjusted ORs calculated with the full cohort of 5395 respondents.

^b^ News sources data only available for 1646 respondents.

In logistic regression models for increase in depressive symptoms, we further identified significant interactions of platform use with age group for Facebook, TikTok, and Snapchat (Table 3). For TikTok and Snapchat, use was associated with depressive symptoms among those ages 35 years or older but not among those younger than age 35 years (eg, Snapchat: aOR, 1.96; 95% CI, 1.44-2.65 vs aOR, 1.17; 95% CI, 0.78-1.77). For Facebook, the opposite pattern was observed; use was associated with depressive symptoms among those younger than 35 years, but not among those aged 35 years and older (aOR, 2.60; 95% CI, 1.46-1.62 vs aOR, 1.12; 95% CI, 0.85-1.48).

**Table 3.** Logistic Regression Models for Association Between Social Media Use at Initial Survey and Clinically Significant Increase in PHQ-9

Platform	Full cohort, OR (95% CI)^a^	*P* value for interaction with age	OR (95% CI)	
			**≥35 y**	**<35 y**
Facebook	1.42 (1.10-1.81)	.04	1.12 (0.85-1.48)	2.60 (1.46-4.62)
Instagram	1.18 (0.95-1.46)	.17	NA	NA
LinkedIn	1.03 (0.80-1.33)	.08	NA	NA
Pinterest	0.94 (0.76-1.16)	.20	NA	NA
TikTok	1.39 (1.03-1.87)	.01	1.67 (1.14-2.45)	1.36 (0.86-2.16)
Twitter	1.05 (0.84-1.31)	.08	NA	NA
Snapchat	1.53 (1.19-1.96)	<.001	1.96 (1.44-2.65)	1.17 (0.78-1.77)
YouTube	1.16 (0.93-1.44)	<.001	1.31 (1.03-1.67)	0.68 (0.41-1.11)

Abbreviations: NA, not applicable; OR, odds ratio; PHQ-9, 9-item Patient Health Questionnaire.

^a^ Cohort includes all participants with PHQ-9 scores less than 5 at first survey who responded to a subsequent survey. Age-stratified analysis was completed only if age-by-platform interaction was nominally significant.

## Discussion

In this analysis of survey data, we found that some forms of social media use—in particular, Snapchat, Facebook, and YouTube—were associated with greater levels of self-reported depressive symptoms on a subsequent survey. Notably, with the exception of Snapchat, this association was not explained in regression models by use of other news sources, suggesting it is not accounted for by differential media consumption more broadly. Likewise, the association was not meaningfully changed by number of social supports or face-to-face social interactions at baseline, suggesting it is not mediated by reduction in social interactions more broadly.

While we cannot test causation, as in a prior study of Facebook discontinuation,^6^ our design allowed us to look at incident self-reported depression following social media use. Our results extend prior observations of elevated depressive symptoms in adolescents^1^ or young adults^2^ associated with social media use in cross-sectional studies. They are also consistent with 2 prior short-term longitudinal studies.^4,5^ Beyond the ability to look longitudinally, this study also extends prior studies by examining a broader age range, with a mean age of 56 years. Our results suggest that the associations previously observed with depressive symptoms are not limited to young adults; indeed, while effect sizes for Facebook are greater among younger adults, effect sizes for TikTok and Snapchat are greater among those age 35 years or older.

### Limitations

This study had several limitations. Results are limited by the inability to control for all potential confounding, lack of dose-response data, and inability to measure nature of social media use, which has been suggested may moderate impact.^8^ Notably, social media use may simply be a marker of underlying vulnerability to depression. On the other hand, one investigation found that viewing one’s own Facebook profile was associated with increased physiological stress response.^9^ The extent to which these findings generalize beyond the COVID-19 pandemic period also remains to be determined; it may be that the effects of social media use are specific to content viewed, for example. A further caveat is that only a (nonrandom) subset of participants return for a second survey, which could yield a biased sample with less generalizability. However, as the surveys are not labeled as COVID-19 specific, the likelihood is lower that these results reflect a selection bias in which individuals with greater interest in, or who had been more affected by, COVID-19 preferentially responded.

## Conclusions

In this survey study of US adults, we identified associations between type of social media use at initial survey and greater levels of depressive symptoms on a subsequent survey. Our findings complement those using cross-sectional designs^2^ or small longitudinal cohorts^4,5^ and extend them to older adults, indicating the need for further investigation of the relationship between social media use and mental health.
